# Collective phases and long-term dynamics in a fish school model with burst-and-coast swimming

**DOI:** 10.1098/rsos.240885

**Published:** 2025-05-09

**Authors:** Weijia Wang, Ramón Escobedo, Stéphane Sanchez, Zhangang Han, Clément Sire, Guy Theraulaz

**Affiliations:** ^1^ Centre de Biologie Intégrative, CNRS, Université de Toulouse III – Paul Sabatier, Centre de Recherches sur la Cognition Animale, Toulouse, France; ^2^ Beijing Normal University School of Systems Science, Beijing, People’s Republic of China; ^3^ Laboratoire de Physique Théorique, Universite Toulouse III Paul Sabatier, Toulouse, France; ^4^ Universidad Carlos III de Madrid Departamento de Matemáticas, Leganés, Community of Madrid, Spain; ^5^ Institut de Recherche en Informatique de Toulouse, Université de Toulouse 1 Capitole UFR Droit et Science Politique, Toulouse, Occitanie, France

**Keywords:** collective motion, intermittent motion, fish school model, agent-based modelling, social interactions

## Abstract

Intermittent and asynchronous burst-and-coast swimming is widely adopted by various species of fish as an energy-efficient mode of locomotion. This swimming mode significantly influences how fish integrate information and make decisions in a social context. Here, we introduce a simplified fish school model in which individuals have an asynchronous burst-and-coast swimming mode and selectively interact only with one or two neighbours that exert the largest influence on their behaviour over a limited spatial range. The interactions consist of a fish that is attracted to and aligned with these neighbours. We show that, by adjusting the interactions between individuals above a sufficiently high level, depending on the relative strength of attraction and alignment, the model can produce a cohesive fish school that replicates the main collective phases observed in nature: schooling, milling and swarming when each individual interacts with only one neighbour; and schooling and swarming when each individual interacts with two neighbours. Moreover, the model showed that these patterns can be maintained over long simulations. However, with the exception of swarming, these patterns do not persist indefinitely, and fish lose cohesion and progressively disperse. We further identified the mechanisms that lead to group dispersion.

## 1. Introduction

Many species of fish living in groups adopt an intermittent ‘burst-and-coast’ swimming mode [1,2]. Swimming is characterized by an active swimming ‘burst’ phase followed by a passive gliding phase and plays many roles in the life of fish, such as energy saving [3–6], decrease in detectability [7] and stabilization of the sensory field [2].

When moving in groups, fish coordinate their swimming through their social interactions: they are both attracted to their neighbours and align with them [8–10]. The form of these social interactions varies between species and depends on the distance between fish, as well as on the orientation and relative position of their neighbours [11–13]. For instance, the range of attraction and alignment interactions has been shown to be much larger in *Hemigrammus rhodostomus* than in *Danio rerio* [14]. Furthermore, several studies have shown that each fish does not interact with all its neighbours but only with a small subset of them [15–17] and, in particular, with those that exert the strongest influence on its movement [18].

The most influential individuals are those who trigger a larger response (larger angle change, i.e. acceleration) at each moment than the other neighbours [19]. This selection reduces the amount of information that must be processed in the brain of the fish and prevents cognitive overload [15,20]. Finally, individuals can modulate the intensity with which they interact with their neighbours according to their physiological state or environmental characteristics [19].

Understanding how the combination of these social inter­actions between fish and their modulation by phy­sio­lo­gical or environmental parameters determines the types and properties of collective movements at the school level is a fundamental question in the research field of collective animal behaviour [21–24]. Most existing fish school models consider individuals to have a continuous swimming mode. Very few models have studied the impact of asynchronous and intermittent movement on swimming coordination [25,26], and even fewer have explored their impact on the resulting collective movement phases [27–30].

In this article, we introduce a general model to study the properties of collective movements of fish schools in which individuals have an asynchronous and intermittent swimming mode. This model is a simplified version of a data-based model originally developed to account for collective swimming behaviour in *H. rhodostomus* [13,18,30]. The model has only nine parameters, including four main parameters for the social interactions (attraction and alignment), describing their intensity and spatial range. The model integrates the intermittent swimming mode of the fish, their asynchronous individual decisions at the onset of the bursting phase and the filtering of social information, which results from the fact that each fish only interacts with, at most, its two most influential neighbours. Compared to other recent burst-and-coast models, the one introduced by Oscar *et al*. [26], which focuses on decision-making via virtual leaders, has 14 parameters and is not suited for large groups in unbounded domains. As for the model introduced by Gyllingberg *et al.* [25], it is limited to two fish and lacks critical individual-scale features such as the exponential fish speed decrease in the gliding phase.

We first describe the model and analyse the different collective phases produced by groups of fish swimming in an unbounded environment for different combinations of the intensity of attraction and alignment interactions. These collective phases correspond to different movement patterns that are adopted by schooling fish, which differ in their level of organization and their collective information processing abilities [22]. We thus distinguish a rather disordered state, which is also called swarming, in which individuals aggregate without cohesion, with a low level of polarization and much more organized states such as schooling, in which individuals are aligned with each other and milling that results from the rotation of fish around an empty core. These forms of collective movement also present adaptive advantages, with swarming improving the initial detection of stimuli, while schooling improves information transfer among fish [31].

We then analyse the long-term behaviour, the stability of each collective phase and the mechanisms by which this stability is eventually lost in the long term. With the exception of a few studies, collective motion models have only been studied on short time scales. Moreover, recent studies have shown that to understand the evolution of biological traits, it is necessary to study the collective behaviour of animal groups over a long time scale (see [32] for a comprehensive review).

We show that this simplified burst-and-coast model in which fish interact asynchronously with only one or two neighbours and within a limited perception range can reproduce the main features of the original data-based model [30], particularly a cohesive fish school and the typical collective phases of swarming, schooling and milling. Moreover, the model shows that these states can be maintained over very long time scales, allowing the identification of the mechanisms by which cohesion is lost in the long term.

## 2. Methods

### 2.1. Fish school model with burst-and-coast swimming

We generalize the model derived from experimental data in [13] and [18] by simplifying fish dynamics and social interaction functions while preserving the essential ingredients of the discrete and asynchronous burst-and-coast swimming mode and the interaction strategy of paying attention to only one or two most influential neighbours.

The ‘burst-and-coast’ swimming mode consists of a successive alternation of sudden acceleration and longer deceleration periods, during which the fish glides along a nearly straight line. These sudden events during which a fish changes both its velocity and its direction of motion are called ‘kicks’, of typical duration 0.1 s, and are interpreted as decision moments. The typical interval between kicks is of order 0.5 s for *H. rhodostomus* [13] but can go up to 1 s and more rarely up to 1.5 s. During the gliding period, the fish moves almost passively, propelled by its inertia resulting from the kick, its speed ultimately decaying quasi-exponentially until the next kick due to the friction exerted by the fluid.

The consecutive kicks of a single fish do not have necessarily the same length and duration. When swimming in groups, the kicks performed by different fish are mostly asynchronous and of different length and duration. The duration of the acceleration phase is typically 5–10 times shorter than that of the gliding phase, and we will therefore assimilate a kick and the corresponding acceleration phase as instantaneous. We will also consider that each fish chooses its direction of motion at the instant of performing a kick, and that it maintains its heading unchanged while decelerating during the gliding phase, resulting in a perfectly straight trajectory between kicks.

The instant of time at which fish i makes its nth kick is denoted by $t_{i}^{n}$, the kick duration by $\tau_{i}^{n}$ and the kick length by $l_{i}^{n}$. The position and ve­lo­ci­ty vectors of fish i at this kicking time are ${\vec{u}}_{i}^{\;n}(x_{i}^{n},y_{i}^{n})$ and ${\vec{v}}_{i}^{\;n}(v_{i,x}^{n},v_{i,y}^{n})$, respectively.

**Figure 1.**
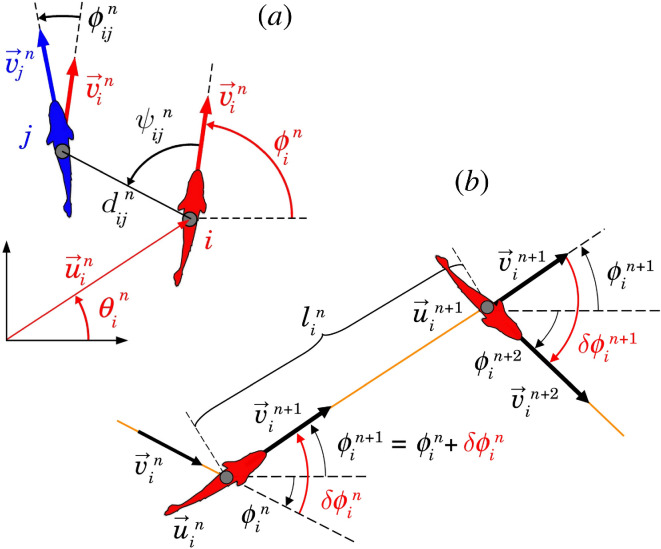
(*a*) Individual (red) and social (black) state variables of fish i with respect to fish j at the instant $t_{i}^{n}$ when fish i performs its nth kick: ${\vec{u}}_{i}^{n}$ and ${\vec{v}}_{i}^{n}$ are the position and velocity vectors of fish i, $\theta_{i}^{n}$ and $\phi_{i}^{n}$ are the angles that these vectors make with the horizontal line, ${\vec{v}}_{j}^{n}$ is the velocity vector of fish j at time $t_{i}^{n}$, $d_{ij}^{n}$ is the distance between fish i and fish j, $\psi_{ij}^{n}$ is the angle with which fish j is perceived by fish i (not necessarily equal to $\psi_{ji}^{n}$), and $\phi_{ij}^{n}=\phi_{j}^{n}-\phi_{i}^{n}$ is the heading difference between both fish (from [30]). (*b*) Schematic of the nth kick performed by fish i moving from ${\vec{u}}_{i}^{n}$ at time $t_{i}^{n}$ to ${\vec{u}}_{i}^{n+1}$ at time $t_{i}^{n+1}$ along a distance $l_{i}^{n}$. Orange lines denote fish trajectory, black wide arrows denote velocity vector, curved arrows represent angles. The heading angle change of fish i at time $t_{i}^{n}$ is $\delta \phi_{i}^{n}$. Fish heading during its nth kick is $\phi_{i}^{n+1}=\phi_{i}^{n}+\delta \phi_{i}^{n}$. Red angles show the heading variation of the fish at the kicking instants $t_{i}^{n}$ and $t_{i}^{n+1}$.

The speed of the fish at the beginning of the kick is given by ${\text{v}}_{i}^{n}=\|{\vec{v}}_{i}^{\;n}\|$. The speed is found experimentally to decay almost exponentially during the gliding phase, with a relaxation time $\tau_{0}$ ($\tau_{0}\approx 0.8$ s for *H. rhodostomus* [13]), while the fish heading orientation $\phi_{i}^{n}=ATAN2(v_{i,y}^{n},v_{i,x}^{n})$ remained unchanged along the nth kick. Positive angles are defined in the counterclockwise direction, with respect to the positive semi-axis of the abscissas of the global system of reference centred in (0,0) (figure 1*a*, variables in red).

The relative state of fish i with respect to fish j is given by $\left(d_{ij},\psi_{ij},\phi_{ij}\right)$*,* where $d_{ij}$ is the distance between them, $\psi_{ij}=\theta_{ij}-\phi_{i}$ is the angle at which fish i perceives fish j, $\theta_{ij}$ is the angle that the vector going from i to j forms with the horizontal line and $\phi_{ij}=\phi_{j}-\phi_{i}$ is the relative heading, which also measures the degree to which i and j are aligned. Note that $\psi_{ij}$ is not necessarily equal to $\psi_{ji}$ (see figure 1*a*, variables in black).

Social interactions are thus described by pairwise interaction functions of attraction and alignment, which scale with the typical body length of fish, and where only the leading mode of the angular components is kept.

Figure 1*b* shows the nth kick performed by fish i at time $t_{i}^{n+1}=t_{i}^{n}+\tau_{i}^{n}$. The position and heading of the fish at the end of the nth kick is given by


(2.1)u→in+1=u→in+τine→(ϕin+1),(2.2)ϕin+1=ϕin+γRgin+∑j∈Jinδϕijn(dijn,ψijn,ϕijn),

where $\vec{e}\;(\phi_{i}^{n+1})$ is the unitary vector pointing in the direction of the angle $\phi_{i}^{n+1}$.

**Figure 2.**
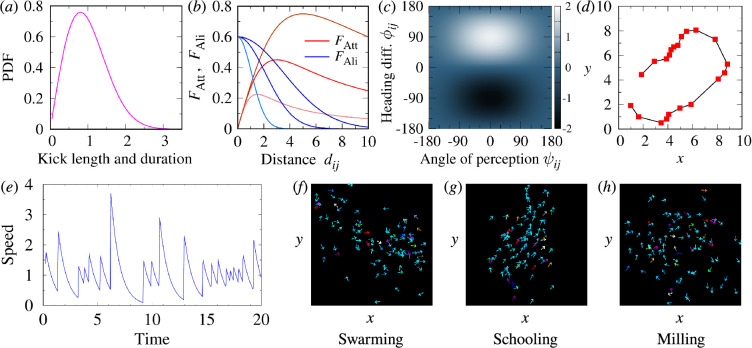
Individual burst-and-coast swimming behaviour, social interaction between fish and collective phases observed in the model. (*a*) Probability density function (PDF) of the kick length and duration. (*b*) Social forces of attraction (red) and alignment (blue) as a function of the distance $d_{ij}$ between fish for three values of the interaction ranges $l_{\text{Att}}=l_{\text{Ali}}=1.5$, 3 and 5. The colour shade is darker as the value is higher. (*c*) Strength of alignment, as the product of an even function of the angle of perception $\psi_{ij}$ and an odd function of the heading difference $\phi_{ij}$. (*d*) Trajectory of a single fish showing the kicking instants (red squares) followed by straight segments (black lines). (*e*) Velocity profile showing the gliding phases corresponding to the 20 kicks shown in (*d*). (*f–h)* Typical collective motion patterns of each phase: (*f*) swarming, (*g*) schooling and (*h*) milling, for k=1. Not all the N=100 fish appear in the figures.

The length and duration of the nth kick of fish i were set equal (unit speed scale), $\tau_{i}^{n}=l_{i}^{n}$, and sampled from a bell-shaped distribution with mean 1 (figure 2*a*).

The heading angle change $\delta \phi_{i}^{n}=\phi_{i}^{n+1}-\phi_{i}^{n}$ of fish i is the result of (i) the random fluctuations of fish $\gamma_{\text{R}}\;g_{i}^{n}$, where $\gamma_{\text{R}}$ is the fluctuation intensity and $g_{i}^{n}$ is a random number sampled from a Gaussian distribution with a mean equal to one; and (ii) the social interactions with other fish $j\in J_{i}^{n}$, where $J_{i}^{n}$ is the set of fish considered to influence the behaviour of fish i.

The *influence* fish j on fish i is precisely determined by the absolute value of the heading angle change of fish i induced by fish j [18],


(2.3) $$I_{ij}(t)=|\delta \phi_{ij}(t)|,$$

and therefore depends on the relative state of both fish. Sorting the neighbours of fish i according to $I_{ij}(t)$ allows the identification of the k=1 or 2 most influential neighbours that constitute the social environment according to the selected social interaction strategy. As shown in our recent results on *H. rhodostomus*, only the two most influential neighbours are required to quantitatively reproduce the behavioural patterns of groups of fish swimming in a circular tank [18]. As the influence of fish on a focal fish can change from one kick to another, the identity of the most influential neighbour(s) can change accordingly.

The strength of the social interaction depends non-linearly on the distance between fish (figure 2*b*) and is modulated by the relative position and heading of both fish (figure 2*c*). Under equal conditions of alignment, fish i will turn right if fish j is on its right side and will turn left otherwise; thus, attraction is an odd function of $\psi_{ij}$. Although experiments with pairs of *H. rhodostomus* have shown that attraction slightly depends on the way fish are aligned with each other, we assume here that the relative heading $\phi_{ij}$ has no effect on attraction strength $\phi_{ij}$: for fish j located at a given place, fish i will turn right if $\phi_{ij}>0$, and will turn left otherwise. Alignment also depends on the relative position of the perceived fish, but in an even way, the alignment force exerted by a neighbour located in front of a fish ($|\psi_{ij}|<90^{\circ }$) is stronger than when the neighbour is located behind ($|\psi_{ij}|>90^{\circ }$), it does not depend on whether the neighbour is located on the right ($\psi_{ij}<0$) or on the left side ($\psi_{ij}>0$) of the focal fish.

Assuming, as in [13], that attraction and alignment are combined in an additive form, and that all contributions can be decoupled as functions of a single state variable, the heading angle change of fish i due to social interaction with one neighbour j is given by


(2.4)δϕij(dij,ψij,ϕij)=γAttdijsin⁡ψij1+(dij/lAtt)2+γAli(1+ϵcos⁡ψij)e−(d/lAli)2sin⁡ϕij,

where all parameters are dimensionless: $\gamma_{\text{Att}}$ and $\gamma_{\text{Ali}}$ are the respective intensities of the attraction and alignment forces, $l_{\text{Att}}$ and $l_{\text{Ali}}$ the respective interaction ranges (scaled with the typical kick length $l_{0}=0.07\;m$), and ϵ is a parameter of anisotropy in the alignment. The first term in equation (2.4) corresponds to the attraction, and the second to the alignment. Both terms have been simplified with respect to the original data-based model [30] by removing the short-range repulsion and alignment and retaining only one Fourier mode in the angular components.

Figure 2*b* shows the strength of the attraction and alignment as a function of the distance between fish for three values of the respective range of interaction, $l_{\text{Att}}$ and $l_{\text{Ali}}$. The modulation of these forces as functions of the angle of perception and the degree of alignment is a simple $\sin\;\psi_{ij}$ for attraction and a two-dimensional function of both angles for the alignment (figure 2*c*).

To calculate $\delta \phi_{ij}$, the relative state of fish j must be known at the time of kicking fish i. As the kicks of different fish are asynchronous, the position and heading angle of fish j must be calculated according to the elapsed time since its last kick. The position of a fish i during the gliding phase following its nth kick, i.e. at time $t_{i}^{n}+s$, $0\leq s\leq \tau_{i}^{n}$, can be written as


(2.5)u→i(tin+s)=u→in+τin1−exp⁡(−s/τ0)1−exp⁡(−τin/τ0)e→(ϕin+1),

where we have used the fact that the fish speed decreases exponentially, with a relaxation time $\tau_{0}$. These intermediate positions also allow a representation of the trajectories of the N fish with a fixed time step.

Figure 2*d*,*e* shows the trajectory of a single fish along 20 kicks and the corresponding speed profile, respectively, illustrating the discrete nature of the burst-and-coast swimming mode. Despite the fact that fish collect information from the environment only at kicking instants, their environment is limited to one or two specific neighbours, their decisions are locked along their kicks and groups of burst-and-coasting fish are able to display highly structured collective behaviours. Figure 2*f*–*h* shows the result of a single run of the model in a large group of N=100 individuals and for three pairs of values of the social interaction parameters, $\left(\gamma_{\text{Att}},\gamma_{\text{Ali}})=(0.6,0.6\right)$, (0.22,0.6) and (0.37,0.2), which give rise to a cohesive school displaying, respectively, a swarming, a schooling and a milling formation.

### 2.2. Quantification of collective behaviour

We characterize the collective behavioural patterns by means of three observables: (i) group cohesion, measured by the dispersion of individuals with respect to the barycentre of the group, (ii) group polarization, measuring how much fish are aligned and (iii) milling index, measuring how much the fish turn around their barycentre as in a vortex formation [13].

The x-coordinates of the position and velocity vectors of the barycentre B are


(2.6) $$\begin{array}{lrr} & x_{B}(t)=\frac{1}{N}\sum_{i=1}^{N}x_{i}(t),\;v_{x}^{B}(t)=\frac{1}{N}\sum_{i=1}^{N}v_{x}^{i}(t), & \end{array}$$

with similar expressions for $y_{B}(t)$ and $v_{y}^{B}(t)$. The heading angle of the barycentre is given by its velocity vector, $\phi_{B}=\text{ATAN2}(v_{y}^{B},v_{x}^{B})$. Then

1. *Group dispersion* is the mean of the square distance of each fish to the barycentre of group B


(2.7) $$\begin{array}{lrr} & D(t)=\sqrt{\frac{1}{N}\sum_{i=1}^{N}\|{\vec{u}}_{i}(t)-{\vec{u}}_{B}(t){\|}^{2}}. & \end{array}$$

Low values of D(t) correspond to highly cohesive groups, whereas high values of D(t) mean that individuals are spatially dispersed. Values of D(t) can become arbitrarily large in a dispersion regime, as the distance between fish increases with the duration of the simulation.

2. *The group polarization* is determined by the fish headings $\phi_{i}$ and independently of the intensity of the speed


(2.8) $$\begin{array}{lrr} & P(t)=\frac{1}{N}\left\|\sum_{i=1}^{N}{\vec{e}}_{i}(t)\right\|, & \end{array}$$

where ${\vec{e}}_{i}={\vec{v}}_{i}/\|{\vec{v}}_{i}\|=(\cos\phi_{i},\sin\phi_{i})$ is the unit vector of fish heading.

Values of P close to 1 indicate that the N individual headings are aligned and point in the same direction; this is what happens when fish swim in a school. Values of P close to 0 indicate that the N vectors point in different directions, but can also mean that vectors are collinear and in opposite directions so that they cancel each other out (e.g. half of the vectors pointing north and the other half pointing south). When the headings are uncorrelated, the polarization index is such that $P∼1/\sqrt{N}$, which becomes small only for large group size N, but which is markedly lower than 1 for any N≥5. For N=100, a value of P≈0.1 would mean that the group is not polarized.

3. *The milling indexM(t)∈[0,1]* measures how much the fish turn in the same direction around their barycentre B,


(2.9)M(t)=|1N∑i=1Nsin⁡(θ¯wi(t))|,

where ${\bar{\theta }}_{\text{w}}^{\;i}(t)={\bar{\phi }}_{i}-{\bar{\theta }}_{i}$. Variables with a bar are defined in the barycentre’s system of reference: ${\bar{x}}_{i}=x_{i}-x_{B}$, ${\bar{v}}_{x}^{i}=v_{x}^{i}-v_{x}^{B}$ (similar expressions for the y-components). Then, the relative position angle and heading of fish i with respect to B are, respectively, ${\bar{\theta }}_{i}=\text{ATAN2}({\bar{y}}_{i},{\bar{x}}_{i})$ and ${\bar{\phi }}_{i}=\text{ATAN2}({\bar{v}}_{y}^{i},{\bar{v}}_{x}^{i})$. Note that ${\bar{\phi }}_{i}\neq \phi_{i}-\phi_{B}$.

Fish rotating counterclockwise (respectively clockwise) around B to make the sum of the bars in equation (2.9) tend towards 1 (respectively −1). The milling index M(t) denotes the intensity of the milling group, regardless of the direction of rotation.

### 2.3. Calculation of the number of groups

The number of groups $N_{\text{G}}$ is calculated with the ‘chain of offspring’ recursive method. We first considered that two fish that are separated by more than a critical distance of interaction do not interact with each other. We tested several critical distances and found that using a distance of four times the interaction range of attraction, $l_{Att}$, the result is in agreement with the direct observation of the spatial configuration. This choice is based on the fact that the attraction interaction reaches its maximum at $l_{Att}$ and decreases thereafter, becoming negligible compared to other factors, such as noise, at a distance larger than $4l_{Att}$. Consequently, an individual fish whose nearest neighbour is further than this critical distance is considered to form a group of size 1. The rest of the groups were then built as follows. Starting with a given fish, add to the group its nearest neighbour (offspring). Then, add the nearest neighbours of this offspring, and so on, until the next nearest neighbour is already in the group. Then, another fish that is not yet in a group is selected, and its ‘family of offspring’ is built with the same recursive procedure.

It may happen that one or more groups are, in fact, at a close distance from one another, or even that a group is located in the interior of another group. In this case, individuals from one group can be subject to the influence of individuals of another group, so that the fish are not really in different groups. To prevent these situations, we merged the groups that are separated by a distance shorter than the minimum interaction range, min$\left\{l_{Att},l_{Ali}\right\}$.

### 2.4. Numerical simulations of the model

The discretization setting to explore the parameter space was chosen to obtain a detailed visualization of the regions of interest and their evolution from one condition to another (large phase diagrams were obtained with $\Delta \gamma_{\text{Att}}=0.005$ and $\Delta \gamma_{\text{Ali}}=0.01$).

**Figure 3.**
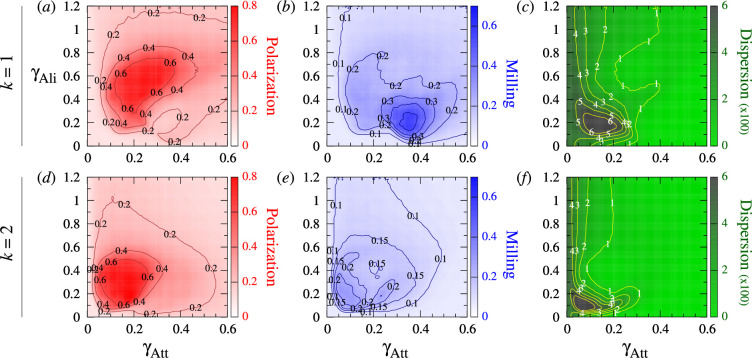
Polarization, milling and dispersion maps for different values of the intensity of attraction and alignment strengths $\gamma_{\text{Att}}$ and $\gamma_{\text{Ali}}$, respectively, when fish interact with their most influential neighbour (k=1) and their two most influential neighbours (k=2). (*ad*) Polarization (red). (*be*) Milling (blue). (*cf*) Dispersion (green). Regions of high colour intensity mean that fish frequently display the characteristic behavioural patterns of schooling, milling or swarming. The green region means that the school of fish is highly cohesive. In the grey region, attraction is too weak and fish quickly disperse. Social interaction ranges are $l_{\text{Att}}=l_{\text{Ali}}=3$m, ϵ=0.8, intensity of random fluctuation (noise) is $\gamma_{\text{R}}=0.2$. Each pixel is the average of 20 runs of 2000 kicks per fish (the first half has been discarded in order to remove the effects of the initial condition). The maps have been smoothed to enhance the representation of level sets.

**Figure 4.**
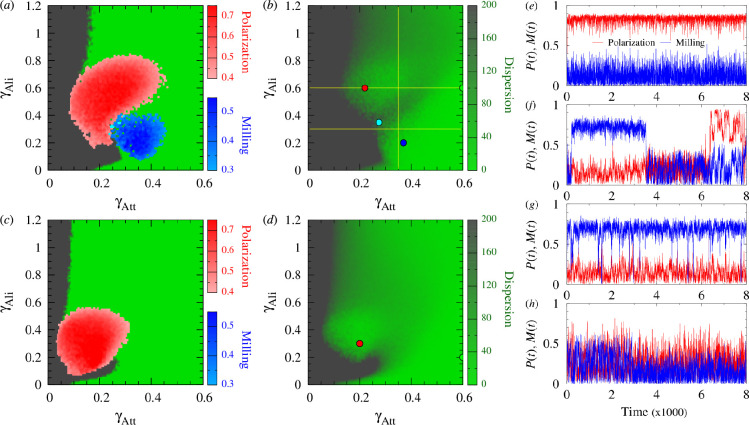
Phase diagrams $\gamma_{\text{Att}}$-$\gamma_{\text{Ali}}$ showing the regions of high polarization (P≥0.4) and high milling (M≥0.3) determining the phases of schooling (red) and milling (blue), respectively, for (*a*) k=1 and (*c*) k=2, superimposed on a binary colour map of dispersion delimiting the swarming phase (D≤200, light green), where the group stays cohesive, and a high dispersion region (D>200, grey). Points where P≥0.4 and M≥0.3 simultaneously are shown in cyan. (*b*) Colour map of dispersion for D∈[0,200] and k=1, and (*d*) for k=2, showing the three transects (yellow lines) at $\gamma_{\text{Att}}=0.35$, $\gamma_{\text{Ali}}=0.6$ and $\gamma_{\text{Ali}}=0.3$, detailed in figure 5. In (*b*) and (*d*), values larger than D=200 appear in dark green. The four coloured points in (*b*) are at (0.22,0.6) (red), (0.37,0.2) (blue), (0.275,0.35) (cyan) and (0.6,0.6) (green). The two points in (*d*) are at (0.2,0.3) (red) and (0.6,0.2) (green). (*e–h*) Time series of polarization P(t) (red lines) and milling M(t) (blue lines) corresponding to the points $\left(\gamma_{\text{Att}},\gamma_{\text{Ali}}\right)$ shown in panel (*b*): (*e*) red point, high polarization and low milling; (*f*) cyan point, alternating high and low values of P(t) and M(t); (*g*) blue point, high milling and low polarization; (*h*) green point, small values of P(t) and M(t). Other parameters are $\gamma_{\text{R}}=0.2$, $l_{\text{Att}}=l_{\text{Ali}}=3$ and ϵ=0.8. Each point of the phase planes is the average of 20 runs of $2\times 10^{4}$ kicks per fish (first half discarded in order to remove the effects of the initial condition). Time series (*e–h*) are extracted from the last instants of one run of $2\times 10^{4}$ kicks per fish.

**Figure 5.**
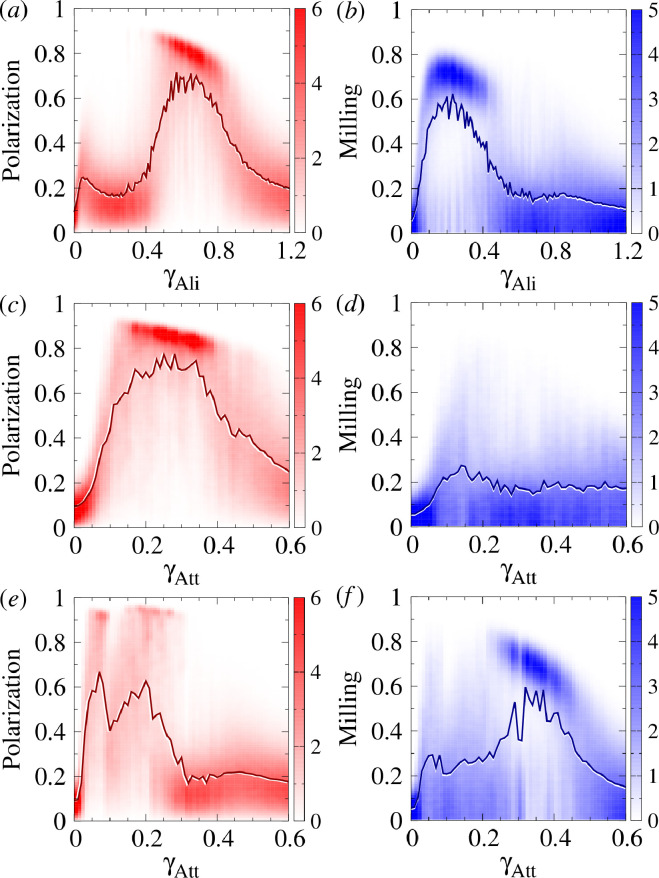
Polarization (red) and milling (blue) probability distribution functions (PDF) and mean values (solid lines) along the transects of the phase diagram for k=1 (yellow lines in figure 4*b*). (*a*,*b*) Vertical transect at $\gamma_{\text{Att}}=0.35$ crossing the three phases, (*c*,*d*) horizontal transect at $\gamma_{\text{Ali}}=0.6$ crossing the polarization and swarming phases and (*e*,*f*) horizontal transect at $\gamma_{\text{Ali}}=0.3$ crossing the milling and swarming phases. In all panels, the variation of colour intensity along the y-axis corresponds to the PDF of the parameter value shown in the x-axis, and solid lines join the corresponding mean value of each PDF. The rest of parameters are $\gamma_{\text{R}}=0.2$, $l_{\text{Att}}=l_{\text{Ali}}=3$, ϵ=0.8 and $\gamma_{\text{R}}=0.2$.

**Figure 6.**
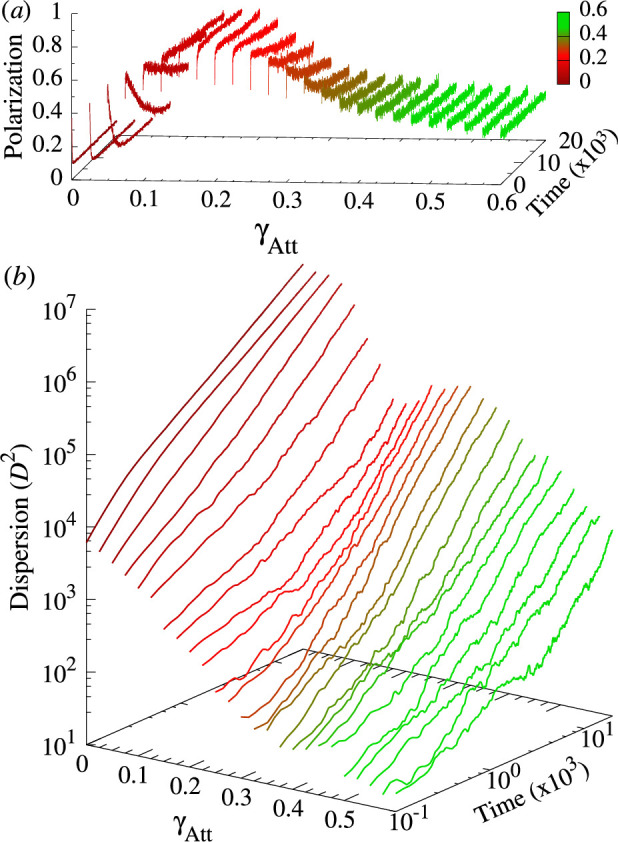
Time series of (*a*) mean polarization P(t) and (*b*) mean squared dispersion $D^{2}(t)$ averaged over 100 long simulations ($2\times 10^{4}$ kicks per fish) for $\gamma_{\text{Ali}}=0.6$, $\gamma_{\text{Att}}\in [0,0.6]$ and k=1 (corresponding to the upper horizontal transect of the phase plane in figure 4*b*). Time and dispersion scales are logarithmic in (*b*). The colours correspond to those used in figure 4*a* to represent the dispersal region and the schooling and swarming phases. Other parameter values: $l_{\text{Att}}=l_{\text{Ali}}=3$, ϵ=0.8, $\gamma_{\text{R}}=0.2$.

**Figure 7.**
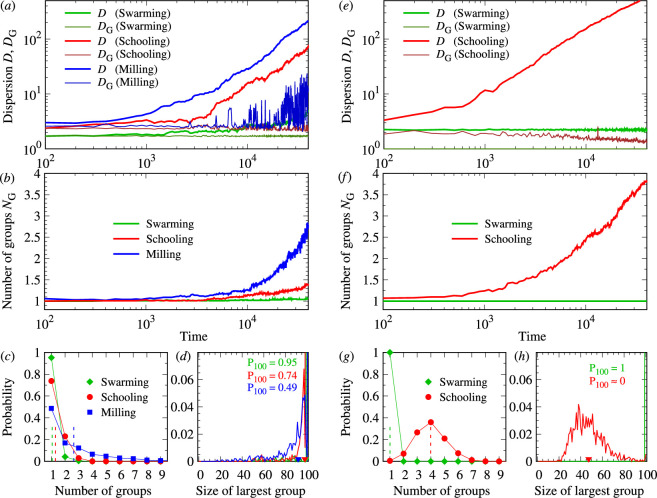
Dispersion, number of groups and size of the largest subgroup for the three points $\left(\gamma_{\text{Att}},\gamma_{\text{Ali}}\right)$ in the swarming (green), schooling (red) and milling (blue) phases shown in figure 4*b*,*d*, for k=1 (*a–d*) and k=2 (*e–h*). (*a*,*e*) Time evolution of the mean dispersion of the whole group D(t) and of the mean dispersion averaged over all subgroups $D_{\text{G}}(t)$ (log-log scale). (*b*,*f*) Time evolution of the mean number of groups $N_{\text{G}}(t)$ (log-normal scale), corresponding to (*a*) and (*e*), respectively. Each line is the average of 1000 runs of $4\times 10^{4}$ kicks per fish. (*c*,*g*) Probability distribution (symbols) and mean value (dashed lines) of the number of groups $N_{\text{G}}$, calculated on the final state of each of the 1000 runs. (*d*,*h*) Probability distribution (solid lines) and mean value (small triangles on the x-axis) of the size of the largest group. The curves of schooling and milling are shifted −1 and +1, respectively, not to overlap the peaks of probability at 100. The range of abscissas is thus extended to [−1,101]. Coloured numbers denote the height of each peak. Parameter values when k=1 are (see the points in figure 4*b*) as follows: swarming: $\left(\gamma_{\text{Att}},\gamma_{\text{Ali}})=(0.6,0.6\right)$, schooling: $\left(\gamma_{\text{Att}},\gamma_{\text{Ali}})=(0.22,0.6\right)$, milling: $\left(\gamma_{\text{Att}},\gamma_{\text{Ali}})=(0.37,0.2\right)$. Parameter values when k=2 are (see the points in figure 4*d*) as follows: swarming: $\left(\gamma_{\text{Att}},\gamma_{\text{Ali}})=(0.6,0.2\right)$, schooling: $\left(\gamma_{\text{Att}},\gamma_{\text{Ali}})=(0.2,0.3\right)$. Initially, fish have the same heading (ϕ=0) and are distributed in an ellipse of long and short half axes a and b, respectively (long axis taken parallel to the x-axis). See the rest of the parameter values in table 1.

The parameter values used in the simulations are shown in table 1*a*. For the colour maps of figure 3, we averaged 20 runs of 2000 kicks per fish. For the phase planes in figure 4, we used 20 runs of a longer duration, $2\times 10^{4}$ kicks per fish. The time series shown in figure 4*e*–*h* were extracted from the last instants of runs of $2\times 10^{4}$ kicks per fish. The probability density function (PDF) in figure 5 is the average of 100 runs, each with 200 kicks per fish. In the long-term analysis, we used the average of 100 runs of $2\times 10^{4}$ kicks per fish in the time series shown in figure 6 and went to an average of up to 1000 runs of $4\times 10^{4}$ kicks per fish for those shown in figure 7. In all cases, we neglected the first half of the time series to remove the effects of the initial condition.

**Table 1.** Parameter values used in the simulations. (a) Interaction strength and range, noise, mean kick duration and length, relaxation time and coefficient of anisotropy; (*b*) long and short axes a and b, respectively, of the ellipse inside which fish are randomly distributed in the initial conditions.

symbol	parameter	values
$\gamma_{\text{Att}}$	attraction strength	0−0.6
$\gamma_{Ali}$	alignment strength	0−1.2
$l_{Att},l_{Ali}$	range of attraction and alignment	3
$\gamma_{R}$	noise intensity	0.2
$\left\langle \tau_{n}\right\rangle ,\left\langle l_{n}\right\rangle$	mean kick duration and length	1
τ0	relaxation time	0.8
ϵ	coefficient of anisotropy	0.8

*K*	phase	*a*	*b*
*k* = 1	swarming	3	1.5
	schooling	5	3
	milling	3	1.5
*k* = 2	swarming	4	2
	schooling	3	1.5
	milling	—	-—

For each run, the initial conditions were sampled from random distributions and were therefore different. Except for the very long simulations in figures 6 and 7, fish are initially randomly distributed in a circle of radius R chosen so that the mean distance between fish, estimated to be approximately $\sqrt{\pi R^{2}/N}$, is of the order of half the range of social interactions, so $R=(l_{\text{Att}}/2)\sqrt{N/\pi }$. The initial headings are sampled from the uniform distribution in $\left(-180^{\circ },180^{\circ }\right)$. For long-term simulations, fish initially have the same heading (ϕ=0) and are distributed in an ellipse of long and short half axes a and b, respectively (the long axis is parallel to the x-axis). The values of a and b for each state and listed k=1 and k=2 are shown in table 1*b*.

## 3. Results

### 3.1. Collective motion patterns and phase diagram

We first explore the parameter space of the model applied to a group of N=100 fish by varying its main parameters, which are the intensity of attraction and alignment, $\gamma_{\text{Att}}$ and $\gamma_{\text{Ali}}$, respectively.

Figure 3 shows the dispersion, polarization and milling, obtained for different values of the intensity of the attraction and alignment $\gamma_{\text{Att}}$ and $\gamma_{\text{Ali}}$ while keeping other parameters fixed and for both social interaction strategies, k=1 and k=2.

When k=1 (figure 3*a*–*c*), the dispersion is uniformly small (D<150) in the right half plane where the attraction strength is large ($\gamma_{Att}>0.3$). For smaller values of $\gamma_{\text{Att}}$, the dispersion is higher and, depending on the alignment strength, reaches very high values (D>600) in a small region where $\gamma_{\text{Att}}\in (0.05,0.25)$ and $\gamma_{\text{Ali}}\in (0.1,0.3)$. Polarization (figure 3*b*) reaches high values (P>0.4 and up to 0.8) in a large kidney-shaped region where $\gamma_{\text{Att}}\in (0.07,0.4)$ and $\gamma_{\text{Att}}\in (0.1,0.8)$. Outside this region, the group is not polarized, P≈0.1−0.2. For N=100 individuals, the expected value of the polarization corresponding to uncorrelated headings is of the order $1/\sqrt{N}=0.1$. Similarly, there is a region of high milling where M>0.3 (up to 0.7 in the centre), located around $\left(\gamma_{\text{Att}},\gamma_{\text{Ali}})=(0.35,0.2\right)$, and whose size is approximately one third of the region of high polarization. Outside this region, the milling was uniform and small (M<0.1). We also observe a slightly visible region of intermediate values of milling (M≈0.2) whose boundary coincides with that of the region of high polarization.

For k=2 (figure 3*d*–*f*), there was no region with high milling. The regions of high polarization and high dispersion are smaller than those for k=1 and are obtained for smaller values of the interaction strengths $\gamma_{\text{Att}}$ and $\gamma_{\text{Ali}}$.

Figure 4*a* shows the collective behavioural phases for the interaction strategy k=1, obtained by super­imposing these regions of high values in a phase diagram. We define the following three phases of collective behaviour:

— Schooling (red): P≥0.4 and M<0.3;
— Milling (blue): P<0.4 and M≥0.3;
— Swarming (green): P<0.4 and M<0.3*.*

We show in cyan the points where P≥0.4 and M≥0.3*.*

Figure 4*b* shows the dispersion with two uniformly coloured regions of low (green) and high (grey) values for k=1. When the dispersion was small, the group remained cohesive at least for the duration of the simulation. The region of high dispersion cannot be considered a collective phase. For k=1 (figure 4*a*,*b*, see also electronic supplementary material, videos S1, S2, S3 and S4), a minimal attraction strength ($\gamma_{\text{Att}}^{\text{min}}\approx 0.09$) is necessary to prevent dispersion. The polarization (red) and milling (blue) phases are mainly located within the low dispersion region, but they also partly cover the region of high dispersion. The polarization and milling phases are located next to each other, with a very small region between (cyan) in which the fish alternate between schooling and milling (figure 4*f*). For higher values of attraction and alignment strengths, there is no more schooling or milling, and the fish adopt a swarming behaviour (green).

For k=2 (figure 4*c*,*d*, see also electronic supplementary material, videos S5 and S6), we first observe that there is no milling phase, meaning that M<0.3 all $\gamma_{\text{Att}}$ and $\gamma_{\text{Ali}}$. The minimal attraction strength to prevent dispersion is smaller than when k=1 ($\gamma_{\text{Att}}^{\text{min}}\approx 0.05$), and the schooling phase is smaller than when k=1. Fish start schooling at considerably smaller values of $\gamma_{\text{Att}}$ (less than half the value leading to schooling when k=1) and $\gamma_{\text{Ali}}$ (less than 2/3 the corresponding value when k=1) and reach higher polarization values. No schooling appears when $\gamma_{\text{Att}}>0.3$ or $\gamma_{\text{Ali}}>0.55$. Similarly, when $\gamma_{\text{Ali}}>0.2$, the swarming phase starts at smaller values of $\gamma_{\text{Att}}$ compared with the condition with k=1. The transition from high to low dispersion was located at half the value of $\gamma_{\text{Att}}$ observed for k=1. In turn, for smaller values of $\gamma_{\text{Ali}}$, the region of dispersion was similar to that observed when k=1, forming a tongue that entered the swarming region up to $\gamma_{\text{Att}}\approx 0.3$. For a very small $\gamma_{\text{Ali}}<0.05$, the swarming phase starts at values of $\gamma_{\text{Att}}$ that are half of those found for k=1 (0.1 instead of 0.2).

Figure 4*e*–*h* shows the time series of polarization and milling for the four points plotted in the phase diagram in figure 4*b* (see also electronic supplementary material, videos S1, S2, S3 and S4). For $\gamma_{\text{Att}}$ and $\gamma_{\text{Ali}}$ picked in the centre of the schooling region, polarization remains almost always above P=0.8 (figure 4*e*). The same occurs for M(t) when $\left(\gamma_{\text{Att}},\gamma_{\text{Ali}}\right)$ are in the centre of the milling phase (figure 4*g*), reaching and maintaining a stable value around M=0.75, although the milling structure is sometimes lost (but quickly recovered) in this example. At the interface of the schooling and milling regions (figure 4*f*), both order parameters were significant on average. This results from intermittent dynamics in which the school can switch back and forth from a polarized to a milling organization. This interfacial region corresponds to large fluctuations in both order parameters and to the strong sensitivity of the group to any external perturbation [33]. Figure 4*h* illustrates the case where $\left(\gamma_{\text{Att}},\gamma_{\text{Ali}}\right)$ are in the swarming phase; even in that case, the group often adopts a spatial configuration that gives rise to non-negligible values of the polarization, up to P=0.6, but only for very short intervals of time. The same occurs with milling (M(t) is often >0.4).

To quantify the extent to which the instantaneous polarization and milling are larger than their respective means, we calculated the PDF of P(t) and M(t) along three transects that cross the behavioural phases of the phase plane (yellow lines in figure 4*b*).

Figure 5 shows that across the behavioural phases of schooling and milling, the corresponding PDF typically exhibits a peak at high values and a long tail, which is responsible for the low mean value. The peak of the PDF of polarization is at approximately P=0.8 and P=0.85 in the two transects crossing the schooling phase, while the mean is at approximately P=0.65 and P=0.73, respectively (figure 5*a*,*c*). Similarly, the peak of the milling is at approximately M=0.7, while the mean is always smaller than M=0.6 (figure 5*b*,*f*).

Outside the schooling and milling phases, both polarization and milling are widely and uniformly distributed around the mean value; see, e.g. $\gamma_{\text{Ali}}\in [0,0.4]$ in figure 5*a* and $\gamma_{\text{Att}}\in [0.3,0.6]$ in figure 5*e* for polarization, and $\gamma_{\text{Ali}}\in [0.5,1.2]$ in figure 5*b* and the whole transect in figure 5*d* for milling. Moreover, the higher the dispersion, the greater the width of the PDF. For $\gamma_{\text{Ali}}\in [0,0.4]$ in figure 5*a*, the dispersion is quite small (D<40) and the PDF of polarization is quite narrow, compared to the interval $\gamma_{\text{Att}}\in [0.4,0.5]$ in figure 5*c*, where the dispersion is higher (D≈80) and the PDF is wider, and with the interval $\gamma_{\text{Att}}\in [0.025,0.275]$ in figure 5*e*, where the dispersion is very high (D>200), and the PDF is practically flat.

The same is true for milling, comparing the interval $\gamma_{\text{Ali}}\in [0.6,1.2]$ in figure 5*b*, where the dispersion is low and the PDF is narrow, with the interval $\gamma_{\text{Att}}\in [0.025,0.275]$ in figure 5*f*, where the dispersion is very high and the PDF is almost flat.

### 3.2. Long-term dynamics and stability of collective phases

Schooling and milling are complex dynamic structures that can be destabilized by many events, leading a few individuals to lose contact with the school and gradually move away from the group, then producing a slow disintegration of the fish school.

In these conditions, very long simulations must be carried out to study the evolution of dispersion and determine how the stability of the spatial structures is lost. Thus, we characterize the stability by monitoring the dispersion of the fish school and the number and size of subgroups along a large number of long simulations (100 runs of $2\times 10^{4}$ kicks per fish), keeping track of the state of the N=100 individuals every 20 kicks, for $\gamma_{\text{Ali}}=0.6$ and $\gamma_{\text{Att}}\in [0,0.6]$ and k=1, which is the upper horizontal transect of the phase plane shown in figure 4*b*.

We used a discretization step $\Delta \gamma_{\text{Att}}=0.025$, attraction and alignment ranges $l_{\text{Att}}=l_{\text{Ali}}=3$, ϵ=0.8, $\gamma_{\text{R}}=0.2$, and an initial condition in which an individual’s position and heading were sampled from a uniform random distribution.

Figure 6 shows the time evolution of the mean polarization and the mean squared dispersion $D^{2}(t)$. As expected, the mean polarization reaches its highest values in the schooling phase, when $\gamma_{\text{Att}}\in (0.15,0.375)$ (red lines). Outside this region, the mean polarization of the school is much smaller for two different reasons, depending on whether $\gamma_{\text{Att}}$ lies in the dispersion region ($\gamma_{\text{Att}}<0.15$) or in the swarming phase ($\gamma_{\text{Att}}>0.375$). When $\gamma_{\text{Att}}<0.15$, the mean squared dispersion quickly increased with time and reached very high values (dark red lines in figure 6*b*). Simultaneously, the mean polarization decreased to the value of random polarization ($P_{0}=0.1$). Furthermore, the smaller $\gamma_{\text{Att}}$, the faster the changes in the polarization and dispersion. In turn, when $\gamma_{\text{Att}}>0.375$ (green lines), the squared dispersion is approximately three decades smaller than that when $\gamma_{\text{Att}}<0.15$, and no decay in time is observed in the average polarization.

This suggests that, for small $\gamma_{\text{Att}}$, polarization is small because the group quickly fragments, and the alignment is not preserved between distant subgroups, even if the alignment is strong enough to allow individuals to align with each other within each subgroup. However, for large values of $\gamma_{\text{Att}}$, the polarization is small because individuals fail to align with each other despite the fact that the group remains cohesive. Indeed, when the attraction becomes too strong, the heading change of the focal fish resulting from the interaction with its most influential neighbour becomes too large and effectively random, leading to a decrease in group polarization.

To verify this, we calculated the time evolution of (i) the mean dispersion D(t) of the N=100 fish, (ii) the number of subgroups $N_{\text{G}}(t)$, (iii) the size of the largest subgroup and (iv) the mean dispersion inside subgroups $D_{\text{G}}(t)$, for values of $\left(\gamma_{\text{Att}},\gamma_{\text{Ali}}\right)$ corresponding to the points shown in each phase of the phase planes of figure 4*b*,*d* for both k=1 and k=2. We used a much larger number of 1000 even longer runs of $4\times 10^{4}$ kicks per fish. Moreover, the initial random heading of the fish can favour the fragments of the group. We consider an initial condition in which all individuals have the same heading and are positioned in an elliptic spatial configuration with an inter-distance similar to that observed when the group is schooling.

Figure 7*a* shows that, for k=1, the dispersion grows in the three phases of schooling, milling and swarming. However, the growth rate was much higher in the schooling and milling phases than in the swarming phase. After t≈5000, dispersion in the milling phase is always half a decade above that observed in the schooling phase and two decades above that observed in the swarming state at the end of the simulations ($t=4\times 10^{4}$). The dispersion in the schooling state is one decade and a half times higher than that observed in the swarming state. The time evolution of the mean number of groups over these 100 runs shows that the high dispersion in the schooling and milling phases is due to fragmentation of the group. Figure 7*a*,*b* shows that the growth rate of the dispersion in the schooling (red) and milling (blue) phases increases precisely when the corresponding number of groups departs from $N_{\text{G}}=1$, at t≈3000 in the schooling case, and sooner, at t≈1000 in the milling phase. In turn, the group remained cohesive in the swarming phase ($N_{\text{G}}\approx 1$ until the final state).

Once separated from the main group, the subgroups behave differently in the schooling and milling phases. Figure 7*a* shows that, in the schooling phase, the mean dispersion inside subgroups remains constant in time ($D_{\text{G}}(t)\approx 2.5$, dark red line in figure 7*a*) and the mean number of subgroups in the final state is very small ($N_{\text{G}}<1.5$, red line in figure 7*b*). However, in the milling case, the mean dispersion inside subgroups continues to grow, especially after t≈10000 ($D_{\text{G}}=10$) at the final state (dark blue line in figure 7*a*), thus inducing new separations and increasing the number of subgroups $N_{\text{G}}$ until the final time (blue line in figure 7*b*).

Figure 7*c*,*d* shows the probability of having a given number of groups in the final configuration and the probability that the largest group has a given size in the final configuration, calculated over 1000 simulations. The fraction of final states in which only one group is present is 95% in the swarming phase, 74% in the schooling phase and 49% in the milling phase. Configurations with the three groups were never found in the swarming phase, and quite rarely in the schooling and milling phases. In all phases, the largest group was very large (mean size larger than 90, figure 7*d*). This means that the typical configuration consists of a single very large group and very few small groups, with no groups of intermediate size. Separation almost never occurs in the swarming phase and is less frequent in the schooling phase than in the milling case, where more subgroups are found.

For k=2, we consider only two points of the phase plane, because there is no milling phase (figure 4*d*). In the swarming phase, the mean dispersion remained at the same value during the entire simulation period (figure 7*e*), and there was only a single group in all final states (figure 7*f*).

In turn, in the schooling case, the dispersion and fragmentation are much higher than those for k=1. The growth rate of the mean dispersion starts to increase much sooner than for k=1, at t≈1000 instead of 4000, and at a similar rate. Therefore, the curve of D(t) is almost half a decade above that observed for k=1 during the second half of the simulations.

Finally, D≈548 when k=2 while D≈71 when k=1. The mean number of groups at the final time is also higher than for k=1, with a peak at $N_{\text{G}}=4$ (figure 7*g*). However, the mean dispersion inside the subgroups remained constant and even slightly decreased (figure 7*e*, dark red curve).

The group remained cohesive in only five of 1000 runs. Final states with five or more are common. Moreover, the largest group is now of moderate size ($G_{1}\approx 35$−55, with a mean value $G_{1}\approx 47.39$, figure 7*h*). This means that when k=2, the dispersion is so fast that the subgroups that separate from the main one are not necessarily small, so that a continuous range of sizes in 20−80 exists at the final state for the largest group, and consequently, a continuous range of small sizes for the smaller subgroups.

## 4. Discussion

We introduced a simplified asynchronous burst-and-coast fish school model to investigate the impact of social interactions and interaction strategies between fish on emerging collective phases and their long-term stability. The dynamics of intermittent and asynchronous swimming modes are essentially discrete in time and space, and this property has a significant impact on the resulting collective states at the group level. Previous studies have shown that the typical gliding distance is an important scale that determines the emergence of group polarization [34], and that intermittent swimming modulates the influence of social interactions between fish on collective states [30]. These properties are closely related to the unique manner in which fish process social information during intermittent swimming, being only sensitive to that information just before the onset of the bursting phase while neglecting it during the gliding phase [13,35]. This type of information processing contrasts with the one classically considered in most fish school models, in which each individual has a smooth speed and continuously updates its direction according to the direction and relative position of the other fish in a defined neighbourhood [36–41].

This simplified model can reproduce the main features of the original data-based model [30]. First, the model shows that social interactions must reach a minimum intensity to maintain a compact school and allow the emergence of coordinated states such as schooling and milling. However, stronger social interactions between fish do not necessarily lead to a higher level of coordination at the group level, and excessively strong social interactions can prevent coordination. As captured by the vertical transect shown in figure 4*b*, for high values of alignment strength, the school completely loses its polarization and its dispersion also slightly increases. This phenomenon does not appear in models with continuous swimming dynamics [9,41], in which the cohesion and polarization of the school increase when the strength of social interactions increases. In fact, when a fish chooses a new heading before performing a kick, the high values of $\gamma_{\text{Att}}$ and $\gamma_{\text{Ali}}$ generate large δϕ in equation (2.2). The variation in headings between fish then increases, ultimately leading to a loss of coherence in their collective movements. When δϕ is even larger, the fish behave like random walkers, and the group gradually disperses.

The model also reproduces the fact that when fish only interact with their most influential neighbour (k=1), the region of the parameter space corresponding to the schooling and milling states is located within a narrow range of values of $\gamma_{\text{Att}}$ and $\gamma_{\text{Ali}}$, which also overlaps the transition region between group dispersal and the swarming state, meaning that collective states are sensitive to small variations in the strength of social interactions.

Moreover, an important result of the model lies in the existence of a bi-stable region in which the fish school alternates between schooling and milling, at the boundary between these two states. Previous studies on species with an intermittent swimming mode, such as rummy-nose tetra (*H. rhodostomus*), zebrafish (*Danio rerio*) and golden shiners (*Notemigonus crysoleucas*), have shown that, when swimming in a group, fish regularly alternate between the schooling and milling states [42–44]. Operating near the transition region between collective states enhances information processing and adaptability in response to environmental cues [33,45–48]. All these studies suggest that these species have the capacity to collectively tune the strength of social interactions (governed by $\gamma_{\text{Att}}$ and $\gamma_{\text{Ali}}$ in the model) such that the system operates at a critical state.

The alternation between schooling and milling in the bi-stable region is spontaneous and induced by random fluctuations of individual behaviour. From the functional point of view, the changes in collective states can also be the result of within-group conflict. As demonstrated by [31], conflicting preferences of individuals for private and social information may contribute to the transition between ordered and disordered states. It would be interesting to study with the model the impact of the heterogeneity of individual sensitivity to social information on collective dynamics.

Finally, the model also shows that the interaction with the two most influential neighbours allows the emergence of coordinated states with lower values of alignment and attraction forces. When k=2, the minimum values of $\gamma_{\text{Att}}$ and $\gamma_{\text{Ali}}$ leading to the schooling state were approximately 2/3 of those found when k=1. However, the region of the phase diagram leading to the schooling state is much smaller than that when k=1 and the region in which milling was found is replaced by swarming. This suggests that the interaction of fish with their two most influential neighbours results in a much stronger effect of social interactions and a reduction in the parameter space, leading to ordered states.

Once the model is validated, we investigate the long-term stability of collective states. The first observation is that these collective states are quite stable, in the sense that they are sustained over a period that far exceeds the time during which environmental conditions can reasonably be expected to remain unchanged in nature. However, for much longer times, cohesion was lost for almost all the cases. Studies have experimentally shown a loss of cohesion in schools of fish over a period of several dozen days [49]. Not only are groups of fish less cohesive over time, but they are also less polarized and swim more slowly. These changes in collective behaviour result from a decrease in the attraction and responsiveness of fish to their neighbours which results from the habituation of fish to their environment in a predator-free context [49,50]. In our model, we considered that the strength of the interactions remained constant so as to study their long-term specific effects independently of any habituation process. The presence of environmental pressures can therefore contribute to strengthening the social interactions between fish and maintaining a high cohesion in schools of fish [49].

When fish interact only with their most influential neighbour (k=1), the fish school loses its overall polarization and milling because of the formation of subgroups. The typical configuration then consists of one major group (of size approx. 90 fish) which coexists with several small groups (figure 7*a*–*d*). In the three states of schooling, milling and swarming, subgroups continue to disperse over the long term (figure 7*a,b*), although dispersion starts before in the milling state and is much slower in the swarming state. When fish interact with their two most influential neighbours (k=2), the stability of the schooling state is reduced, and there is no milling state. In contrast, the swarming state remains cohesive. This is a consequence of the stronger social influence of fish.

The emergence of subgroups resulting in a maximal typical size for cohesive groups can be biologically relevant for a given species. On the other hand, in our model based on the interaction with a very few of the most influential neighbours, larger groups could be stabilized by having individuals also interact with the coarse-grained density, for instance, by being also attracted towards the centre of mass of the group. In the future, we plan to study such models coupling the notion of most influential neighbours to a coarse-grained attractive interaction, corresponding to a filtering of the information at the local and global scales, respectively.

In summary, this study provides a comprehensive understanding of the collective dynamics of large fish schools in which individuals perform burst-and-coast swimming. We have shown that in this simplified asynchronous burst-and-coast fish school model, both the strength of social interactions and interaction strategies between fish determine the type of collective state. In particular, the strategy that consists of each fish only interacting with its most influential neighbours (i.e. when k=1) allows the school to display coordinated states over a much larger range of attraction and alignment parameters than when k=2, while still promoting stability of the schooling state over the long term. Finally, an analysis of the long-term dynamics of the schooling, milling and swarming states revealed the typical mode of dispersion that reduces the stability of these states.

## Data Availability

The code for all the simulations is available at: https://figshare.com/s/63777e150997c9a1700a. Supplementary material is available online [51].
